# Book Reviews

**Published:** 1799-09

**Authors:** 


### PRACTICE OF MEDICINE AND SURGERY.

*A Treatise on Bilious Diseases and Indigestion; with the effects of Quassy and Natron in those disorders:* By J. Gibson, *M. D.* Surgeon in the Royal Navy, &c. 8vo. 68 pp. 1799. (Two Shillings.) *London;* Murray and Highley.

Every medical practitioner is apprised of the frequency and distressing nature of dyspeptic complaints, whether arising from an idiopathic disease in the stomach itself, from a derangement in the biliary or pancreatic secretions, or in a secondary way from more general diseases of the system, such as gout, pregnancy, fever, &c.

The success in the cure will always depend upon assigning the true seat and cause of the evil, and then applying the most efficacious remedies.

Our author's treatise is confined to the latter class of means. His favourite remedy is—

℞. Ligni quassiæ incis. sesquidrach.

Natron drach. ij. cum semisse

Aquæ fervent. lb. ij.—Infunde per horam & cola.

Dosis cochl. iv. mag. ter quarterve de die.

He occasionally increases the sal sodæ, and sometimes gives calomel, salts, soap, and aloes, camphor and ipecacuanha, steel and myrrh, and warm water.

He does not, however, exclusively confine himself to dyspepsia, but introduces jaundice, dry belly-ach, ague, cholera morbus, &c. as depending on the bile.

---

*Observations deduced from Facts and Experiments, tending to evince the non-existence of Typhus-contagion: interspersed with remarks on animal life, and on*

*on those laws by which it is governed; also with some remarks on the nature of those diseases, which are epidemic at sea.* By J. Frank, Surgeon in his Majesty's navy, &c. 8vo. 70 pp. 1779. *London;* Johnson.

At a time when the nature of febrile contagion, and the most effectual means of preventing its ravages, engage so much attention through the whole continent of America, and among the physicians of the British navy and army in Europe, our readers will doubtless be surprised to find a pupil of Blane, Robertson, and Trotter in England, and an eminent physician at Calcutta*, attempting to prove, from their own observation and experience, that *such contagion* has no existence.

Dr. Mitchill, and his numerous adherents in America, appear to support their opinion, by referring the cause of their endemics to a particular state of the atmosphere. Even Dr. Robertson, who imputes all idiopathic fever to contagion, says, page 88: "Whatever has a tendency to debilitate the system, may either be a remote or a proximate cause of fever, according to the constitution of the patients," &c. See our Number V. p. 474.

It is not our province to give decisive opinions; but, by suggesting arguments, facts, and experiments, to enable the reader to form just ideas for himself: we have, however, no hesitation in admitting the importance of the subject.

---

*Practical Observations on the Cure of Wounds and Ulcers on the Legs, with and without rest; illustrated with Cases:* By Thomas Whately, &c.

[Concluded from our last Number, pp. 88—91.]

With respect to the *manner* of applying the roller, Mr. Whately is anxious to give the most pointed and unequivocal directions; and as we have already quoted some of the particulars relative to this subject, we think it our duty to extract the succeeding part of the "Postscript," in the words of the ingenious author; and thus enable the reader to form a just opinion on a subject of great importance in chirurgical practice.

"In applying a roller, the first circle should be made *round* the *lowest* part of the ancles as near as possible to the heel; the second should be formed from thence round the foot; the third should be passed again round the foot quite to the toes. The roller should then be passed from the foot round the ancles and instep a second time, to make the fourth circle. In doing this it should be brought nearer (but not over) the point of the heel than it was at the *first time* of going round this part, The fifth circle should pass over the ancles again, and not more than half an inch higher up the leg than the fourth circle. The sixth, seventh, eighth and ninth circles should ascend spirally along the small of the leg, at the *exact distance* of three fourths of an inch from each other. Having proceeded thus far up the leg, we may begin to increase the distances of the circles from each other; they may succeed each other upward to the knee, at the distance of from one to two inches, according to the size and shape of the leg. At that part where the calf of the leg commences, it is generally necessary to let the upper edge of the roller be once, twice, or thrice, turned downwards for about half the circumference of the leg, in order to make the roller lay smooth between the middle of the calf, and the small of the leg. When the roller has been thus applied as far as the knee, there will be a portion of it to spare of perhaps a yard in length; this remainder should be brought down by spiral windings, at greater distances

---

* Dr. Maclean.

tances from each other than thofe which were made on the afcent of the roller The windings fhould in general be completed in the fmall of the leg, where the roller fhould be pinned.

"In *many* cafes it is neceffary to apply the roller *over the heel.* Where this is done, the firft circle fhould be made as low as poffible round the ancles; as in the former defcription. From thence, the fecond circle of the roller fhould pafs from the inftep over one fide of the heel, and be brought over the other fide of the heel to the inftep again. The third circle fhould be paffed round the ancles a fecond time, but ftill nearer to the heel than the firft circle was. The roller fhould after this be brought back to the foot, and paffed round it to make the fourth circle. A fifth circle fhould be again made (though it is not in all cafes abfolutely neceffary) round the foot, to the toes. To make the fixth circle, the roller fhould be brought back, and paffed round the ancles again. The feventh, eighth, ninth, tenth, and eleventh circles fhould afcend fpirally at the *exact diftance* of three fourths of an inch from each other; thefe diftances commencing at the fixth circle. The roller fhould then be carried to the knee, and be brought down again to the fmall of the leg, as defcribed in the former inftruction.

"In applying the compreffes, it is neceffary in every inftance to put them on one by one, and not all in a mafs, though they be of a proper fize and number. They fhould be croffed in different directions; the largeft of them fhould in no cafe be longer than juft to meet on the oppofite fide of the leg to which they are applied. I have in many inftances feen the compreffes applied by patients of fuch a length as to go round the leg like a roller, and be faftened together with pins. This method generally wrinkles and blifters the fkin, and by no means anfwers the purpofe of making a compreffion on the part where it is moft wanted. I never fuffer a pin to be ufed in the compreffes. If the fame compreffes in any cafe be applied two days together they fhould be always turned on the contrary fide at each re-application, in order to prevent wrinkles on the fkin.

"I muft now reply to two objections made by Mr. Baynton, in the 39th page of the fecond edition of his work. The firft is, that it is difficult to retain the roller on the parts to which it is applied; the fecond is, that it gives pain to the patient. In anfwer to the firft of thefe objections, my experience warrants me to fay that a flannel roller will in almoft every inftance keep the exact pofition it was firft placed in for much longer time than is neceffary. I have feen thefe rollers many hundred times keep their fituations without any variation whatever for two days; and that too without the leaft reftraint upon exercife. This has happened in thofe cafes, where from the diftance of the patient, or from the circumftance of his being nearly cured, I have wifhed to drefs the leg only every forty-eight hours. I muft go a ftep further, and obferve, that I have feen repeated inftances in which thefe rollers have remained in their fituation for three or four days, and even nearly for a week without being applied afrefh. In fhort, it is one of the beft properties of a flannel roller, that it is eafily retained in its fituation, when well applied. In *every* inftance in which it is neceffary to ufe one, I could pledge myfelf to apply it in fuch a manner, as fhould prevent its altering its pofition for two days. The method I fhould ufe I have already defcribed; in addition to which nothing more would be neceffary, even in thofe cafes where the fhape of the leg is peculiarly unfavourable to the retention of a bandage, than the infertion of a few pins.

"In anfwer to the fecond objection, I obferve, that I have invariably found, that when a flannel roller has been applied in the manner here defcribed, and has not been drawn unneceffarily tight, it gives no pain. It fits nearly as as easy as a common stocking, and allows a very free motion and exercise of the limb. It has been stated in this work, that the application of the compresses makes the necessary degree of pressure on the ulcer, and thereby prevents the necessity of drawing the roller so tight over the other parts of the leg, as would have been necessary were the compresses not used.

"There is another circumstance which Mr. Baynton considers as giving his method a great advantage over the roller, which is, that by means of the plaster, the edges of the sore may be made to approximate in such a manner that the cicatrix, or new-formed skin, will be less after a cure performed by this method, than by any other. In almost all these cases, before the cure is attempted the leg is more or less enlarged by swelling; and as this swelling is entirely removed by compression, it readily allows the skin to approximate on the healing of an ulcer. Added to this, there is a process of Nature always going on in healing an ulcer or wound in any part of the body (whether there be a loss of substance or not), by which a cicatrix is always considerably less than the previous size of the sore. This effect occurs in all cases, whether the patient be cured by the horizontal position, a roller, or by strips of adhesive plaster. The size of this cicatrix will likewise vary in different cases where the ulcers have been of the same size, by whichever of these three methods they be cured. It will be larger in those ulcers which are accompanied with strong adhesions of the adjoining parts, than in those where such adhesions have not been produced; and this effect will take place to the greatest degree where the ulcers are situated over the tibia, and by long continuance have produced immoveable adhesions of the cellular substance to the adjoining periosteum. The adhesive plaster, when applied as a bandage, will without doubt, leave as small a cicatrix as any other method of cure; but for the reasons already assigned, I do not believe, that the cicatrx will in any case be *smaller* than that produced by a roller. In every case cured by the latter method, I have found the cicatrix very small, when compared with the previous size of the ulcer."

---

*Descriptive Account of a New Method of treating old Ulcers of the Legs:* By THOMAS BAYNTON, &c.

[Concluded from our last Number, pp. 92 and 93.]

In furnishing the reader with an account of the method adopted by Mr. Baynton, in the treatment of old ulcers of the legs, we were, for want of room in our last Journal, reluctantly obliged to discontinue the quotations from the author's treatise, immediately before we mentioned his explanation of the *modus operandi*, and the respectable testimonies he has adduced in favour of his practice.—At present, therefore, we resume the subject, from that part of his work where he has described the mode of applying the bandage, and managing the dressings.

"If the parts be much inflamed," continues Mr. Baynton, "or the discharges very profuse, they should be well moistened and kept cool with cold spring water, poured upon them as often as the heat may indicate to be necessary, or perhaps at least once every hour. The patient may take what exercise he pleases, and it will be always found, that an alleviation of his pain, and the promotion of his cure, will follow as its consequence, though, under other modes of treating the disease, it aggravates the pain, and prevents the cure.

"These means, when it can be made convenient, should be applied soon after rising in the morning, as the legs of persons affected with this disease are then found most free from tumefaction, and the advantages will be greater greater than when they are applied to limbs in a fwollen ftate. But at whatever time the applications be made, or in whatever condition the parts be found, I believe it will always happen, that cures may be obtained by thefe means alone, except in one fpecies of the difeafe, which feldom occurs, but that will be hereafter defcribed. The firft application will fometimes occafion pain, which, however, fubfides in a fhort time, and is felt lefs fenfibly at every fucceeding dreffing. The force with which the ends are drawn over the limb, muft then be gradually increafed, and when the parts are reftored to their natural ftate of eafe and fenfibility, which will foon happen, as much may be applied as the callico will bear, or the furgeon can exert; efpecially if the limb be in that enlarged and incompreffible ftate, which has been denominated the fcorbutic, or if the edges of the wound be widely feparated from each other.

"It was obferved in the preceding part of this Treatife, that I feared the confequence of breaking the fkin in the vicinity of the fores; later experience has proved fuch occurrences to be of no confequence on any part except the tendon Achilles, thofe wounds being always healed again in a few days; whereas on the tendon, fuch accidents occafion more trouble, and require fometimes the care of many weeks. I therefore now make it a practice, wherever the cafe requires confiderable extenfion of the fkin upon that part of the limb, efpecially if the patient be of a fpare habit, to defend the tendon with a fmall fhred of foft leather, previoufly to the application of the adhefive flips.

"It may be neceffary to add, that cures will be generally obtained without difficulty, by the mere application of the flips and bandage, but when the parts are much inflamed, the fecretions great, or the feafon hot, the frequent application of cold water will be found a valuable auxiliary, and may be always fafely had recourfe to, where the heat of the parts is greater than is natural, and the body free from perfpiration."

After having illuftrated and exemplified the fuperior advantages of his practice by eighteen cafes, the laft of which deferves particular notice, as the fwelling, pain, and inflammation of the leg arofe from weaknefs of the abforbents, occafioned by a fracture of one of the bones of the leg, the author concludes his account with the following judicious remarks:

"In many other inftances of weaknefs and lamenefs, which fucceeded to fractures of the bones and ftrains of the joints, the adhefive plafters prepared and applied, fo as to form bandages, have proved very ufeful, through the fupport they afforded; and I believe it will be generally found, that the confinement required in fuch cafes may be confiderably diminifhed, and frequently rendered unneceffary, by their application. This fhould, perhaps, be expected from what has been fuggefted concerning the theory of their effects; but if it be conjectured, that the fame advantages may be obtained by common bandages, my perfonal experience, as well as the reports of my patients, unfortunately enable me to affert the contrary, as a contufion and ftrain of one of my knees, and a fimilar injury of my left ancle, the former received in the month of May laft, and the latter about ten days fince, by the falling of horfes, prove that it is poffible to walk without much inconvenience, when the parts are fo fupported, at a time that it is fcarcely poffible to ftand, if they receive only the fupport of a common roller.

"Thefe facts fo ftrengthen fome of the opinions delivered concerning the *manner* whereby the cure of many ulcers is accomplifhed, that it would be improper to omit their infertion. The practical interference will not, I believe, be found lefs deferving of attention, as it informs us that plafters denominated denominated strengthening, prove so in consequence of their affording a *peculiar kind* of support to the systems of vessels, rather than through the qualities of their ingredients; and that they become much more effectual when applied so as to encompass and support the whole, or part of a diseased limb, than when *merely placed* upon it, with the expectation of advantages from the properties of their component parts."

---

*An entire new Treatise on Leeches; wherein the nature, properties, and uses of that animal are clearly explained.* By G. HORN, Apothecary, &c. 8vo. 30 pp. 1s. 6d. *London;* Symonds.

The importance of this valuable *instrumentum medicinæ* will doubtless induce the public to inquire into its uses, and the best means of its propagation and preservation, for the purposes of medicine and surgery; on all which subjects our author appears to have taken considerable pains to convey information.

